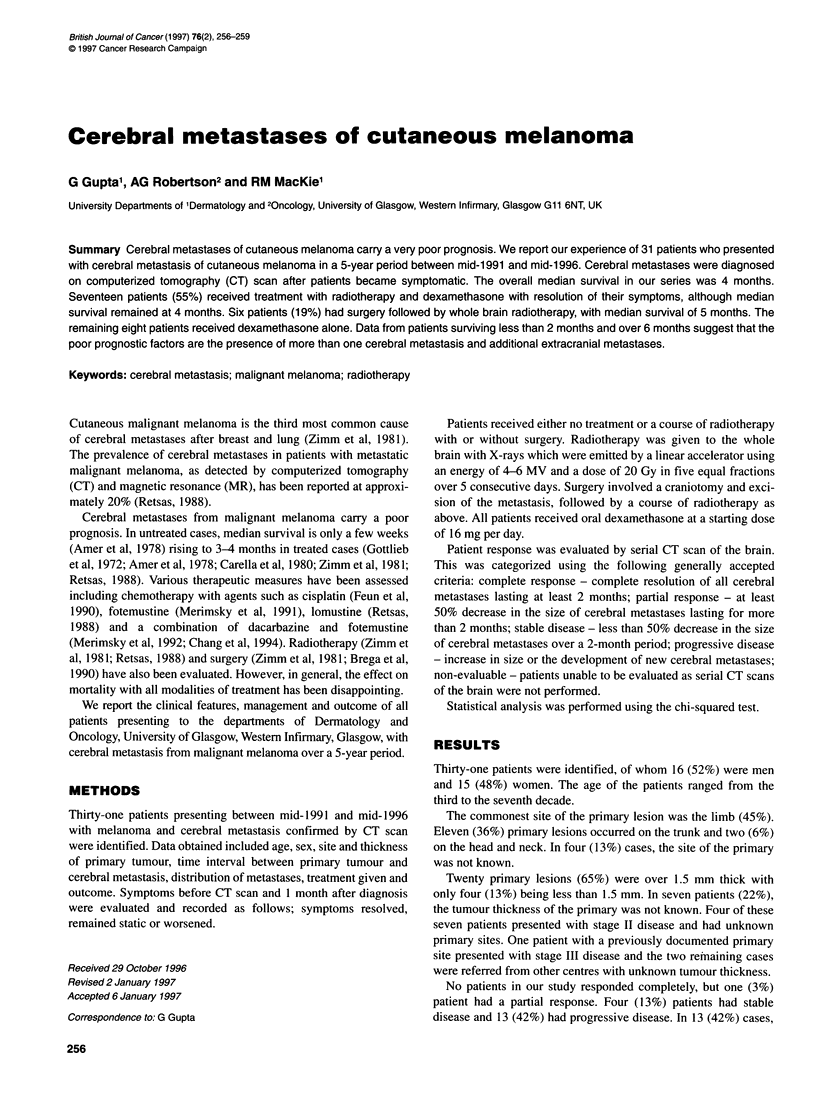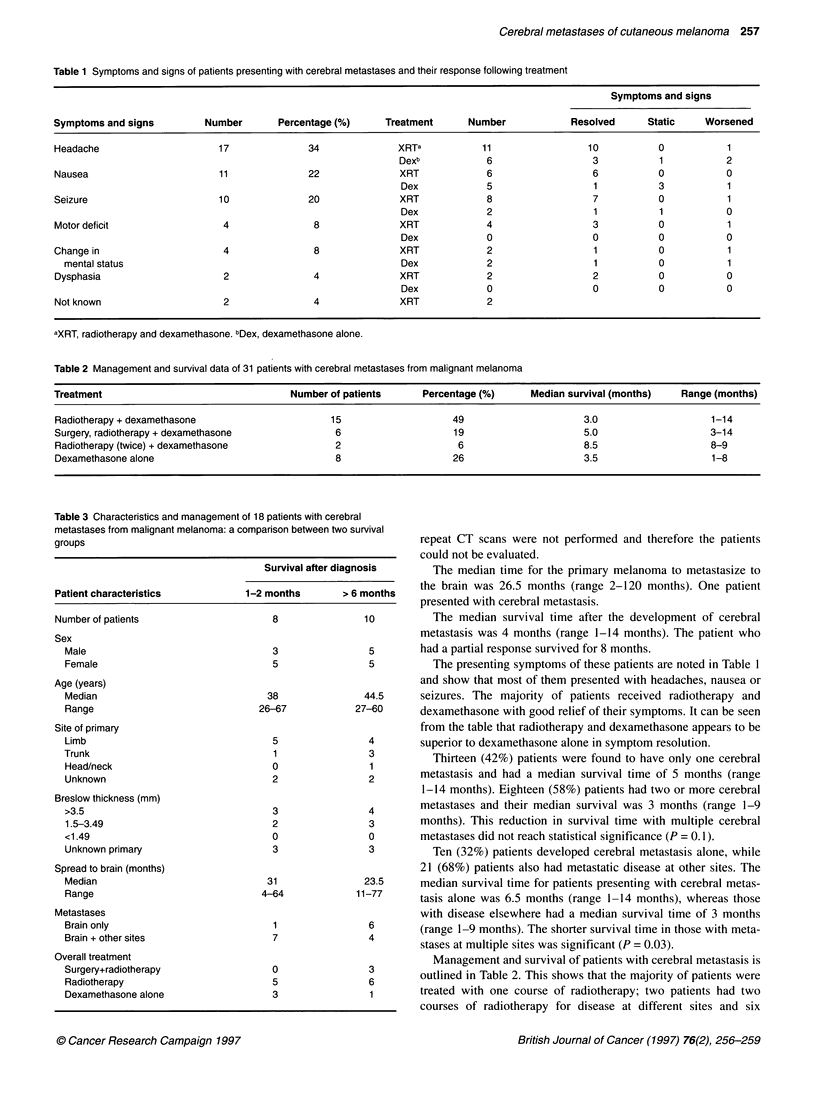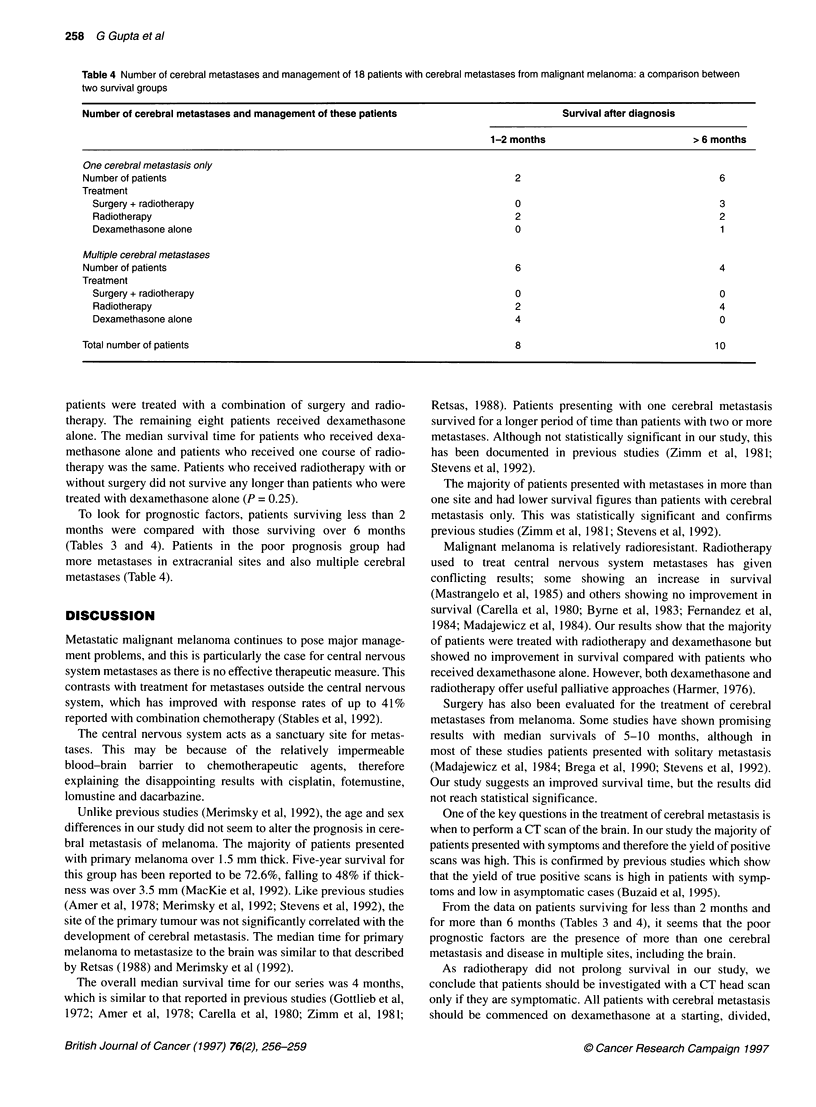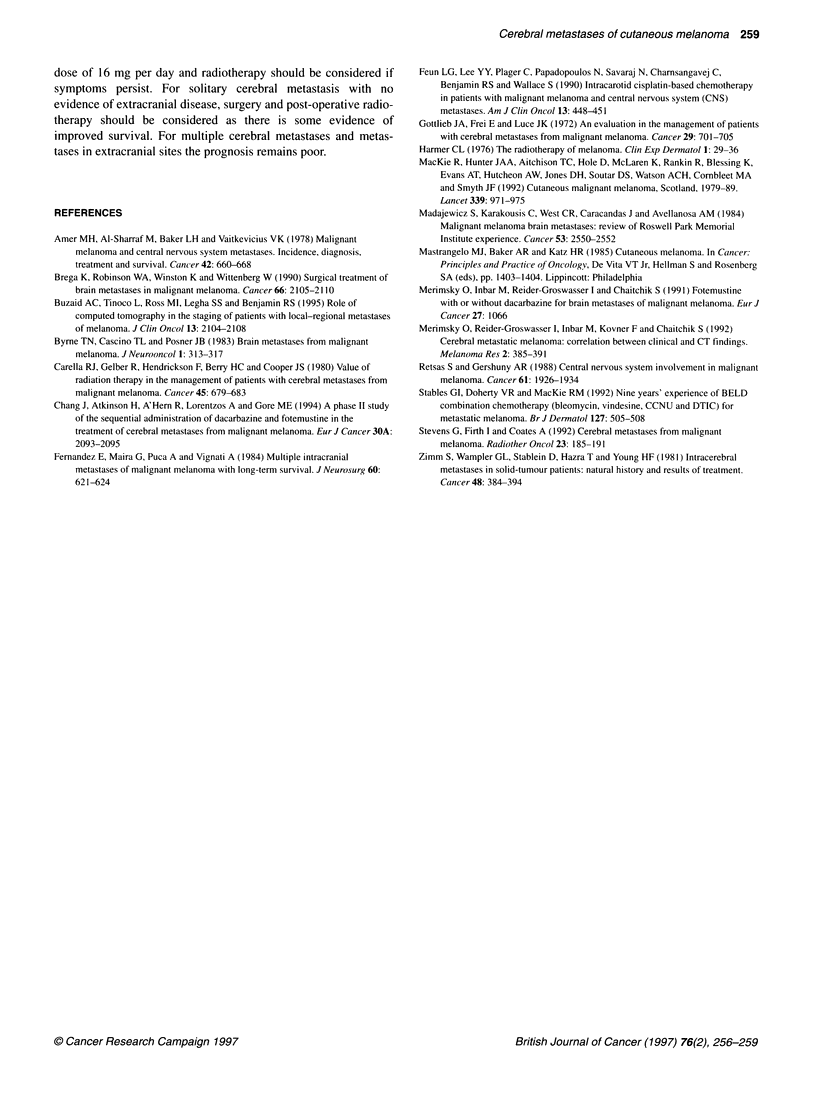# Cerebral metastases of cutaneous melanoma.

**DOI:** 10.1038/bjc.1997.371

**Published:** 1997

**Authors:** G. Gupta, A. G. Robertson, R. M. MacKie

**Affiliations:** University Department of Dermatology, University of Glasgow, Western Infirmary, UK.

## Abstract

Cerebral metastases of cutaneous melanoma carry a very poor prognosis. We report our experience of 31 patients who presented with cerebral metastasis of cutaneous melanoma in a 5-year period between mid-1991 and mid-1996. Cerebral metastases were diagnosed on computerized tomography (CT) scan after patients became symptomatic. The overall median survival in our series was 4 months. Seventeen patients (55%) received treatment with radiotherapy and dexamethasone with resolution of their symptoms, although median survival remained at 4 months. Six patients (19%) had surgery followed by whole brain radiotherapy, with median survival of 5 months. The remaining eight patients received dexamethasone alone. Data from patients surviving less than 2 months and over 6 months suggest that the poor prognostic factors are the presence of more than one cerebral metastasis and additional extracranial metastases.


British Journal of Cancer (1997) 76(2), 256–259
© 1997 Cancer Research Campaign

### Cerebral metastases of cutaneous melanoma

G Gupta[1], AG Robertson[2] and RM MacKie[1]

University Departments of [1]Dermatology and [2]Oncology, University of Glasgow, Western Infirmary, Glasgow G11 6NT, UK

**Summary** Cerebral metastases of cutaneous melanoma carry a very poor prognosis. We report our experience of 31 patients who presented with cerebral metastasis of cutaneous melanoma in a 5-year period between mid-1991 and mid-1996. Cerebral metastases were diagnosed on computerized tomography (CT) scan after patients became symptomatic. The overall median survival in our series was 4 months. Seventeen patients (55%) received treatment with radiotherapy and dexamethasone with resolution of their symptoms, although median survival remained at 4 months. Six patients (19%) had surgery followed by whole brain radiotherapy, with median survival of 5 months. The remaining eight patients received dexamethasone alone. Data from patients surviving less than 2 months and over 6 months suggest that the poor prognostic factors are the presence of more than one cerebral metastasis and additional extracranial metastases.

**Keywords:** cerebral metastasis; malignant melanoma; radiotherapy

Cutaneous malignant melanoma is the third most common cause of cerebral metastases after breast and lung (Zimm et al, 1981). The prevalence of cerebral metastases in patients with metastatic malignant melanoma, as detected by computerized tomography (CT) and magnetic resonance (MR), has been reported at approximately 20% (Retsas, 1988).

Cerebral metastases from malignant melanoma carry a poor prognosis. In untreated cases, median survival is only a few weeks (Amer et al, 1978) rising to 3–4 months in treated cases (Gottlieb et al, 1972; Amer et al, 1978; Carella et al, 1980; Zimm et al, 1981; Retsas, 1988). Various therapeutic measures have been assessed including chemotherapy with agents such as cisplatin (Feun et al, 1990), fotemustine (Merimsky et al, 1991), lomustine (Retsas, 1988) and a combination of dacarbazine and fotemustine (Merimsky et al, 1992; Chang et al, 1994). Radiotherapy (Zimm et al, 1981; Retsas, 1988) and surgery (Zimm et al, 1981; Brega et al, 1990) have also been evaluated. However, in general, the effect on mortality with all modalities of treatment has been disappointing.

We report the clinical features, management and outcome of all patients presenting to the departments of Dermatology and Oncology, University of Glasgow, Western Infirmary, Glasgow, with cerebral metastasis from malignant melanoma over a 5-year period.

### METHODS

Thirty-one patients presenting between mid-1991 and mid-1996 with melanoma and cerebral metastasis confirmed by CT scan were identified. Data obtained included age, sex, site and thickness of primary tumour, time interval between primary tumour and cerebral metastasis, distribution of metastases, treatment given and outcome. Symptoms before CT scan and 1 month after diagnosis were evaluated and recorded as follows; symptoms resolved, remained static or worsened.

*Received 29 October 1996*
*Revised 2 January 1997*
*Accepted 6 January 1997*

*Correspondence to:* G Gupta

Patients received either no treatment or a course of radiotherapy with or without surgery. Radiotherapy was given to the whole brain with X-rays which were emitted by a linear accelerator using an energy of 4–6 MV and a dose of 20 Gy in five equal fractions over 5 consecutive days. Surgery involved a craniotomy and excision of the metastasis, followed by a course of radiotherapy as above. All patients received oral dexamethasone at a starting dose of 16 mg per day.

Patient response was evaluated by serial CT scan of the brain. This was categorized using the following generally accepted criteria: complete response – complete resolution of all cerebral metastases lasting at least 2 months; partial response – at least 50% decrease in the size of cerebral metastases lasting for more than 2 months; stable disease – less than 50% decrease in the size of cerebral metastases over a 2-month period; progressive disease – increase in size or the development of new cerebral metastases; non-evaluable – patients unable to be evaluated as serial CT scans of the brain were not performed.

Statistical analysis was performed using the chi-squared test.

### RESULTS

Thirty-one patients were identified, of whom 16 (52%) were men and 15 (48%) women. The age of the patients ranged from the third to the seventh decade.

The commonest site of the primary lesion was the limb (45%). Eleven (36%) primary lesions occurred on the trunk and two (6%) on the head and neck. In four (13%) cases, the site of the primary was not known.

Twenty primary lesions (65%) were over 1.5 mm thick with only four (13%) being less than 1.5 mm. In seven patients (22%), the tumour thickness of the primary was not known. Four of these seven patients presented with stage II disease and had unknown primary sites. One patient with a previously documented primary site presented with stage III disease and the two remaining cases were referred from other centres with unknown tumour thickness.

No patients in our study responded completely, but one (3%) patient had a partial response. Four (13%) patients had stable disease and 13 (42%) had progressive disease. In 13 (42%) cases,

**Table 1** Symptoms and signs of patients presenting with cerebral metastases and their response following treatment

| | | | | | Symptoms and signs | | |
|---|---|---|---|---|---|---|---|
| **Symptoms and signs** | **Number** | **Percentage (%)** | **Treatment** | **Number** | **Resolved** | **Static** | **Worsened** |
| Headache | 17 | 34 | XRT[a] | 11 | 10 | 0 | 1 |
| | | | Dex[b] | 6 | 3 | 1 | 2 |
| Nausea | 11 | 22 | XRT | 6 | 6 | 0 | 0 |
| | | | Dex | 5 | 1 | 3 | 1 |
| Seizure | 10 | 20 | XRT | 8 | 7 | 0 | 1 |
| | | | Dex | 2 | 1 | 1 | 0 |
| Motor deficit | 4 | 8 | XRT | 4 | 3 | 0 | 1 |
| | | | Dex | 0 | 0 | 0 | 0 |
| Change in mental status | 4 | 8 | XRT | 2 | 1 | 0 | 1 |
| | | | Dex | 2 | 1 | 0 | 1 |
| Dysphasia | 2 | 4 | XRT | 2 | 2 | 0 | 0 |
| | | | Dex | 0 | 0 | 0 | 0 |
| Not known | 2 | 4 | XRT | 2 | | | |

[a]XRT, radiotherapy and dexamethasone. [b]Dex, dexamethasone alone.

**Table 2** Management and survival data of 31 patients with cerebral metastases from malignant melanoma

| **Treatment** | **Number of patients** | **Percentage (%)** | **Median survival (months)** | **Range (months)** |
|---|---|---|---|---|
| Radiotherapy + dexamethasone | 15 | 49 | 3.0 | 1–14 |
| Surgery, radiotherapy + dexamethasone | 6 | 19 | 5.0 | 3–14 |
| Radiotherapy (twice) + dexamethasone | 2 | 6 | 8.5 | 8–9 |
| Dexamethasone alone | 8 | 26 | 3.5 | 1–8 |

**Table 3** Characteristics and management of 18 patients with cerebral metastases from malignant melanoma: a comparison between two survival groups

| | Survival after diagnosis | |
|---|---|---|
| **Patient characteristics** | **1–2 months** | **> 6 months** |
| Number of patients | 8 | 10 |
| Sex | | |
| Male | 3 | 5 |
| Female | 5 | 5 |
| Age (years) | | |
| Median | 38 | 44.5 |
| Range | 26–67 | 27–60 |
| Site of primary | | |
| Limb | 5 | 4 |
| Trunk | 1 | 3 |
| Head/neck | 0 | 1 |
| Unknown | 2 | 2 |
| Breslow thickness (mm) | | |
| >3.5 | 3 | 4 |
| 1.5–3.49 | 2 | 3 |
| <1.49 | 0 | 0 |
| Unknown primary | 3 | 3 |
| Spread to brain (months) | | |
| Median | 31 | 23.5 |
| Range | 4–64 | 11–77 |
| Metastases | | |
| Brain only | 1 | 6 |
| Brain + other sites | 7 | 4 |
| Overall treatment | | |
| Surgery+radiotherapy | 0 | 3 |
| Radiotherapy | 5 | 6 |
| Dexamethasone alone | 3 | 1 |

repeat CT scans were not performed and therefore the patients could not be evaluated.

The median time for the primary melanoma to metastasize to the brain was 26.5 months (range 2–120 months). One patient presented with cerebral metastasis.

The median survival time after the development of cerebral metastasis was 4 months (range 1–14 months). The patient who had a partial response survived for 8 months.

The presenting symptoms of these patients are noted in Table 1 and show that most of them presented with headaches, nausea or seizures. The majority of patients received radiotherapy and dexamethasone with good relief of their symptoms. It can be seen from the table that radiotherapy and dexamethasone appears to be superior to dexamethasone alone in symptom resolution.

Thirteen (42%) patients were found to have only one cerebral metastasis and had a median survival time of 5 months (range 1–14 months). Eighteen (58%) patients had two or more cerebral metastases and their median survival was 3 months (range 1–9 months). This reduction in survival time with multiple cerebral metastases did not reach statistical significance ($P = 0.1$).

Ten (32%) patients developed cerebral metastasis alone, while 21 (68%) patients also had metastatic disease at other sites. The median survival time for patients presenting with cerebral metastasis alone was 6.5 months (range 1–14 months), whereas those with disease elsewhere had a median survival time of 3 months (range 1–9 months). The shorter survival time in those with metastases at multiple sites was significant ($P = 0.03$).

Management and survival of patients with cerebral metastasis is outlined in Table 2. This shows that the majority of patients were treated with one course of radiotherapy; two patients had two courses of radiotherapy for disease at different sites and six

© Cancer Research Campaign 1997

**Table 4** Number of cerebral metastases and management of 18 patients with cerebral metastases from malignant melanoma: a comparison between two survival groups

| Number of cerebral metastases and management of these patients | Survival after diagnosis | |
|---|---|---|
| | 1–2 months | > 6 months |
| *One cerebral metastasis only* | | |
| Number of patients | 2 | 6 |
| Treatment | | |
| Surgery + radiotherapy | 0 | 3 |
| Radiotherapy | 2 | 2 |
| Dexamethasone alone | 0 | 1 |
| *Multiple cerebral metastases* | | |
| Number of patients | 6 | 4 |
| Treatment | | |
| Surgery + radiotherapy | 0 | 0 |
| Radiotherapy | 2 | 4 |
| Dexamethasone alone | 4 | 0 |
| Total number of patients | 8 | 10 |

patients were treated with a combination of surgery and radiotherapy. The remaining eight patients received dexamethasone alone. The median survival time for patients who received dexamethasone alone and patients who received one course of radiotherapy was the same. Patients who received radiotherapy with or without surgery did not survive any longer than patients who were treated with dexamethasone alone ($P = 0.25$).

To look for prognostic factors, patients surviving less than 2 months were compared with those surviving over 6 months (Tables 3 and 4). Patients in the poor prognosis group had more metastases in extracranial sites and also multiple cerebral metastases (Table 4).

### DISCUSSION

Metastatic malignant melanoma continues to pose major management problems, and this is particularly the case for central nervous system metastases as there is no effective therapeutic measure. This contrasts with treatment for metastases outside the central nervous system, which has improved with response rates of up to 41% reported with combination chemotherapy (Stables et al, 1992).

The central nervous system acts as a sanctuary site for metastases. This may be because of the relatively impermeable blood–brain barrier to chemotherapeutic agents, therefore explaining the disappointing results with cisplatin, fotemustine, lomustine and dacarbazine.

Unlike previous studies (Merimsky et al, 1992), the age and sex differences in our study did not seem to alter the prognosis in cerebral metastasis of melanoma. The majority of patients presented with primary melanoma over 1.5 mm thick. Five-year survival for this group has been reported to be 72.6%, falling to 48% if thickness was over 3.5 mm (MacKie et al, 1992). Like previous studies (Amer et al, 1978; Merimsky et al, 1992; Stevens et al, 1992), the site of the primary tumour was not significantly correlated with the development of cerebral metastasis. The median time for primary melanoma to metastasize to the brain was similar to that described by Retsas (1988) and Merimsky et al (1992).

The overall median survival time for our series was 4 months, which is similar to that reported in previous studies (Gottlieb et al, 1972; Amer et al, 1978; Carella et al, 1980; Zimm et al, 1981; Retsas, 1988). Patients presenting with one cerebral metastasis survived for a longer period of time than patients with two or more metastases. Although not statistically significant in our study, this has been documented in previous studies (Zimm et al, 1981; Stevens et al, 1992).

The majority of patients presented with metastases in more than one site and had lower survival figures than patients with cerebral metastasis only. This was statistically significant and confirms previous studies (Zimm et al, 1981; Stevens et al, 1992).

Malignant melanoma is relatively radioresistant. Radiotherapy used to treat central nervous system metastases has given conflicting results; some showing an increase in survival (Mastrangelo et al, 1985) and others showing no improvement in survival (Carella et al, 1980; Byrne et al, 1983; Fernandez et al, 1984; Madajewicz et al, 1984). Our results show that the majority of patients were treated with radiotherapy and dexamethasone but showed no improvement in survival compared with patients who received dexamethasone alone. However, both dexamethasone and radiotherapy offer useful palliative approaches (Harmer, 1976).

Surgery has also been evaluated for the treatment of cerebral metastases from melanoma. Some studies have shown promising results with median survivals of 5–10 months, although in most of these studies patients presented with solitary metastasis (Madajewicz et al, 1984; Brega et al, 1990; Stevens et al, 1992). Our study suggests an improved survival time, but the results did not reach statistical significance.

One of the key questions in the treatment of cerebral metastasis is when to perform a CT scan of the brain. In our study the majority of patients presented with symptoms and therefore the yield of positive scans was high. This is confirmed by previous studies which show that the yield of true positive scans is high in patients with symptoms and low in asymptomatic cases (Buzaid et al, 1995).

From the data on patients surviving for less than 2 months and for more than 6 months (Tables 3 and 4), it seems that the poor prognostic factors are the presence of more than one cerebral metastasis and disease in multiple sites, including the brain.

As radiotherapy did not prolong survival in our study, we conclude that patients should be investigated with a CT head scan only if they are symptomatic. All patients with cerebral metastasis should be commenced on dexamethasone at a starting, divided,

© Cancer Research Campaign 1997

dose of 16 mg per day and radiotherapy should be considered if symptoms persist. For solitary cerebral metastasis with no evidence of extracranial disease, surgery and post-operative radiotherapy should be considered as there is some evidence of improved survival. For multiple cerebral metastases and metastases in extracranial sites the prognosis remains poor.

### REFERENCES

Amer MH, Al-Sharraf M, Baker LH and Vaitkevicius VK (1978) Malignant melanoma and central nervous system metastases. Incidence, diagnosis, treatment and survival. *Cancer* **42**: 660–668

Brega K, Robinson WA, Winston K and Wittenberg W (1990) Surgical treatment of brain metastases in malignant melanoma. *Cancer* **66**: 2105–2110

Buzaid AC, Tinoco L, Ross MI, Legha SS and Benjamin RS (1995) Role of computed tomography in the staging of patients with local–regional metastases of melanoma. *J Clin Oncol* **13**: 2104–2108

Byrne TN, Cascino TL and Posner JB (1983) Brain metastases from malignant melanoma. *J Neurooncol* **1**: 313–317

Carella RJ, Gelber R, Hendrickson F, Berry HC and Cooper JS (1980) Value of radiation therapy in the management of patients with cerebral metastases from malignant melanoma. *Cancer* **45**: 679–683

Chang J, Atkinson H, A'Hern R, Lorentzos A and Gore ME (1994) A phase II study of the sequential administration of dacarbazine and fotemustine in the treatment of cerebral metastases from malignant melanoma. *Eur J Cancer* **30A**: 2093–2095

Fernandez E, Maira G, Puca A and Vignati A (1984) Multiple intracranial metastases of malignant melanoma with long-term survival. *J Neurosurg* **60**: 621–624

Feun LG, Lee YY, Plager C, Papadopoulos N, Savaraj N, Charnsangavej C, Benjamin RS and Wallace S (1990) Intracarotid cisplatin-based chemotherapy in patients with malignant melanoma and central nervous system (CNS) metastases. *Am J Clin Oncol* **13**: 448–451

Gottlieb JA, Frei E and Luce JK (1972) An evaluation in the management of patients with cerebral metastases from malignant melanoma. *Cancer* **29**: 701–705

Harmer CL (1976) The radiotherapy of melanoma. *Clin Exp Dermatol* **1**: 29–36

MacKie R, Hunter JAA, Aitchison TC, Hole D, McLaren K, Rankin R, Blessing K, Evans AT, Hutcheon AW, Jones DH, Soutar DS, Watson ACH, Cornbleet MA and Smyth JF (1992) Cutaneous malignant melanoma, Scotland, 1979–89. *Lancet* **339**: 971–975

Madajewicz S, Karakousis C, West CR, Caracandas J and Avellanosa AM (1984) Malignant melanoma brain metastases: review of Roswell Park Memorial Institute experience. *Cancer* **53**: 2550–2552

Mastrangelo MJ, Baker AR and Katz HR (1985) Cutaneous melanoma. In *Cancer: Principles and Practice of Oncology*, De Vita VT Jr, Hellman S and Rosenberg SA (eds), pp. 1403–1404. Lippincott: Philadelphia

Merimsky O, Inbar M, Reider-Groswasser I and Chaitchik S (1991) Fotemustine with or without dacarbazine for brain metastases of malignant melanoma. *Eur J Cancer* **27**: 1066

Merimsky O, Reider-Groswasser I, Inbar M, Kovner F and Chaitchik S (1992) Cerebral metastatic melanoma: correlation between clinical and CT findings. *Melanoma Res* **2**: 385–391

Retsas S and Gershuny AR (1988) Central nervous system involvement in malignant melanoma. *Cancer* **61**: 1926–1934

Stables GI, Doherty VR and MacKie RM (1992) Nine years' experience of BELD combination chemotherapy (bleomycin, vindesine, CCNU and DTIC) for metastatic melanoma. *Br J Dermatol* **127**: 505–508

Stevens G, Firth I and Coates A (1992) Cerebral metastases from malignant melanoma. *Radiother Oncol* **23**: 185–191

Zimm S, Wampler GL, Stablein D, Hazra T and Young HF (1981) Intracerebral metastases in solid-tumour patients: natural history and results of treatment. *Cancer* **48**: 384–394

© Cancer Research Campaign 1997